# Probiotics, prebiotics, antibiotic, Chinese herbal medicine, and fecal microbiota transplantation in irritable bowel syndrome: Protocol for a systematic review and network meta-analysis

**DOI:** 10.1097/MD.0000000000021502

**Published:** 2020-08-07

**Authors:** Ying He, Rui Xu, Wei Wang, Jie Zhang, Xiaoyu Hu

**Affiliations:** a Hospital of Chengdu University of Traditional Chinese Medicine, Chengdu city, Sichuan province; b Beijing University of Chinese Medicine, Beijing city, China.

**Keywords:** antibiotic, Chinese herbal medicine, fecal microbiota transplantation, network meta-analysis, prebiotics, probiotics, protocol

## Abstract

**Background:**

Irritable bowel syndrome (IBS) is a functional gastrointestinal disease, with a high global incidence, which seriously influences the quality of life and work efficiency of patients. Extensive research showed that IBS is related to changes in the intestinal microenvironment. The novel treatment strategy targeting the gut microbiota is being actively implemented. Probiotics, antibiotics, prebiotics, fecal microbiota transplantation, and Chinese Herbal Medicine have been proven to be effective in the treatment of IBS, and all have an impact on the intestinal flora of patients. However, these 5 treatments have their own pros and cons and have not been systematically evaluated and compared. Therefore, this study will indirectly compare the safety and effectiveness of these 5 methods in the treatment of IBS through network meta-analysis.

**Methods:**

The following databases including Embase, Pubmed, Cochrane Central Register of Controlled Trials, Chinese Biomedical Literature Database, WHO International Clinical Trials Registry Platform and ClinicalTrials.gov will be retrieved from inception to June 2020 without language restrictions. Literature selection, data extraction, and bias analysis will be done by 2 researchers. The primary outcome is global symptoms improvement. The secondary outcomes will include individual IBS symptom scores, emotional response, and adverse events. The conventional pair-wise meta-analysis will be performed using Stata V.14.0 and be pooled using a random-effects model. We will use WinBUGS V.1.4.3 (Cambridge, United Kingdom) with a Bayesian hierarchical random-effects model to conduct the network meta-analysis.

**Results:**

This study will provide systematic reviews and indirect network comparison results about treatments of IBS.

**Conclusions:**

This study will systematically evaluate and compare 5 intestinal flora-related therapies for IBS and to provide an evidence-based medical decision-making basis for clinicians.

**Trial registration number:**

INPLASY202050047

## 1 Introduction

Irritable bowel syndrome (IBS) is a type of functional gastrointestinal disorders (FGIDs), characterized by recurrent abdominal pain, which is associated with defecation or accompanied by changes in bowel habits.^[1]^ IBS is a frequent FGIDs with a global prevalence of approximately 10%, and the prevalence is lowest in Southeast Asia (7%), while in South America, the incidence is about 3 times that of South Asia (21%).^[2]^ In Europe, the prevalence of IBS with constipation (IBS-C) or IBS with a mixed bowel pattern (IBC-M) may be higher than IBS with diarrhea (IBS-D).^[3]^ The symptoms of patients vary with each subtype, but there is no doubt that IBS will seriously affect patients’ quality of life and work efficiency, and increase social medical expenditure.^[4]^ The etiology and pathogenesis of IBS remain unknown. Treatment strategies target symptoms only cannot help the patients to recover and back to normal life.^[5]^ According to the recommendations of Rome IV treatments of IBS mainly include peripheral drugs, systemic drugs, microbial/immunomodulatory therapy, and complementary and alternative medicine (CAM). Accumulating evidence suggests that IBS is closely related to the change of intestinal microorganisms. Studies have shown a negative correlation between abdominal pain and the abundance of *Bifidobacteria* in the intestinal cavity.^[6]^ Based on this, treatment strategies targeting the intestinal flora are being actively implemented in IBS. The definition of microbial therapy in Roman IV mainly includes the following categories: probiotics, antibiotics, prebiotics, and special diets. Probiotics are defined as living microorganisms that confer a health benefit to the host, lactic acid bacteria, and *Bifidobacteria* are the most prevalent used probiotics.^[7,8]^ Up to date, probiotics have been widely used to treat gastrointestinal diseases.^[9]^ Results of a double-blind, randomized placebo-controlled trial suggest that probiotic supplementation can relieve clinical symptoms in patients with IBS.^[10]^ Prebiotics is a nondigestible food ingredient that stimulates the growth of favorable microbial strains in the gut, thereby benefiting the host.^[11]^ The effectiveness of antibiotics in the treatment of IBS has long been confirmed. Rifaximin has played an important role in the treatment of IBS as a non-oral absorption spectrum antibiotic. Rifaximin can reduce the overall symptoms and bloating symptoms of IBS patients and the efficacy is significantly better than the placebo.^[12,13]^ However, the use of antibiotics will induce an imbalance of intestinal bacterial flora, increasing the risk to develop antibiotic-associated diarrhea caused by C. difficile infection.^[14,15]^ Among the recommended treatments for IBS in Roman IV, fecal microbiota transplantation (FMT) is considered a very promising treatment and is successfully used to treat IBS.^[16]^ FMT can achieve the therapeutic effect through the restoration of a healthy microbiota.^[17]^ CAM is of great significance in the treatment of IBS. Almost 30% to 50% of patients with FGIDs have received CAM treatment.^[18,19]^ As the most representative type of CAM therapy, CHM has accumulated rich experience in the treatment of IBS. Studies have shown that CHM can effectively improve IBS symptoms without significantly increasing adverse reactions.^[20]^ There are a variety of chemical ingredients in CHM including rich polysaccharides and glycosides, which are regarded as a natural prebiotic.^[21]^ Studies have shown that as major active components of ginseng, polysaccharide can greatly restore the intestinal flora.^[22]^ Due to the important role of natural medicines in the treatment of IBS and its special regulation function on the intestinal flora, CHM should be included in the analysis of IBS microecological adjustment treatment methods to make the study more comprehensive.

With the in-depth study of intestinal flora, the importance of intestinal flora has been more and more confirmed. All the 5 treatment schemes mentioned above can have an impact on the intestinal flora of patients. However, these 5 treatment methods all have their own advantages and disadvantages, so far there is no direct comparison between them. Therefore, this study will conduct an network meta-analysis (NMA) to indirectly compare the safety and efficacy of these 5 methods in the treatment of IBS, and select the optimal scheme for the treatment of IBS, which will provide scientific evidence for clinicians in the selection of treatment strategies for IBS.

## 2 Methods

### 2.1 Protocol register

This study is a systematic review and meta-analysis of interventional studies on IBS. The final results will be reported according to the guidelines of Preferred Reporting Items for network meta-analyses (PRISMA-NMA).^[23]^ The study protocol will be drafted according to the recommendations listed in the Preferred Reporting Items for Systematic Reviews and Meta-Analyses for Protocols (PRISMA-P).^[24]^ This protocol has been registered on the INPLASY platform (https://inplasy.com/) with an assigned registration number INPLASY202050047.

### 2.2 Ethics and dissemination

This is a re-study of the results of existing research. Subjects were not directly included for analysis, so ethical approval is not needed for this study. The findings will be disseminated through conference presentations, media, and peer-reviewed journals.

### 2.3 Inclusion and exclusion criteria

#### 2.3.1 Types of study

Randomized controlled trials.

#### 2.3.2 Participants

We will include studies in which the diagnostic criteria for IBS patients must meet Manning criteria, Rome I, Rome II, Rome III, Rome IV criteria, or the Kruis score. Subjects are adults aged ≥ 18 years.

#### 2.3.3 Interventions

Treatment strategies include the following 5: Oral CHM treatment under the guidance of syndrome differentiation, and no restrictions on dosage forms, probiotics, prebiotics, antibiotics, fecal transplantation via oral capsule administration. All 5 methods listed above can be used as monotherapy or combined treatments, and the minimum treatment duration is 7 days. Controlled interventions will include a placebo or another intervention in the five treatments mentioned above.

#### 2.3.4 Outcome measures

Primary outcomes will include efficacy assessments of global symptoms cure or improvement from baseline to end of study. The secondary outcomes will include the effect of therapy on individual IBS symptom scores (abdominal pain, distension, and urgency); Emotional response; The number of adverse events and withdrawal due to adverse events. In recent years, various instruments are available for the assessment of IBS symptoms. We will not limit the types of IBS symptoms evaluation criteria in this systematic review. Outcome measurement information is preferably reported by the patient, but if this is unavailable then as assessed by a physician or data obtained through questionnaires are acceptable.^[25]^ Studies that do not meet the inclusion criteria or that are difficult to extract data from will be excluded.

### 2.4 Literature search strategy

The following electronic databases will be searched: Embase, Pubmed, Cochrane Central Register of Controlled Trials, and Chinese Biomedical Literature Database. We will also search other trial databases, including WHO International Clinical Trials Registry Platform (apps. who.int/ trialsearch/), and ClinicalTrials.gov (www.clinicaltrials.gov/) to collect studies with results but not yet published and to find other supplementary information of the included trials. The search strategy will include medical subject headings (MeSH) and keywords associated with prebiotics, probiotics, antibiotics, FMT, and CHM in the treatment of IBS. All databases will be searched from inception to June 2020 without language restrictions. Besides, reference lists of all retrieved articles especially related review articles will be also manually examined. The example of search strategy for PubMed is shown in Table 1.

**Table 1:**
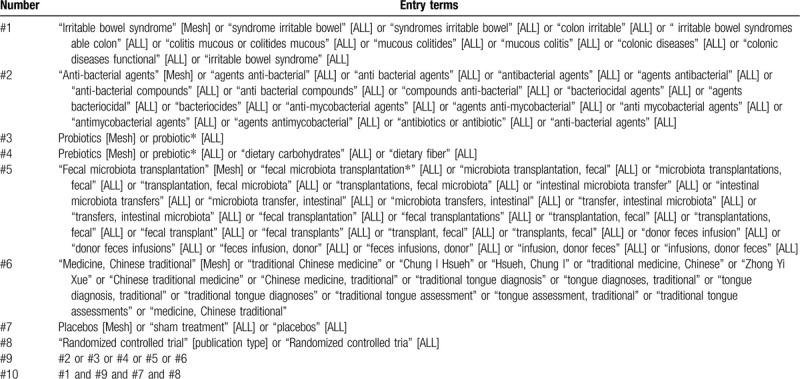
Search strategy in PubMed.

### 2.5 Selection of literatures

**Figure 1:**
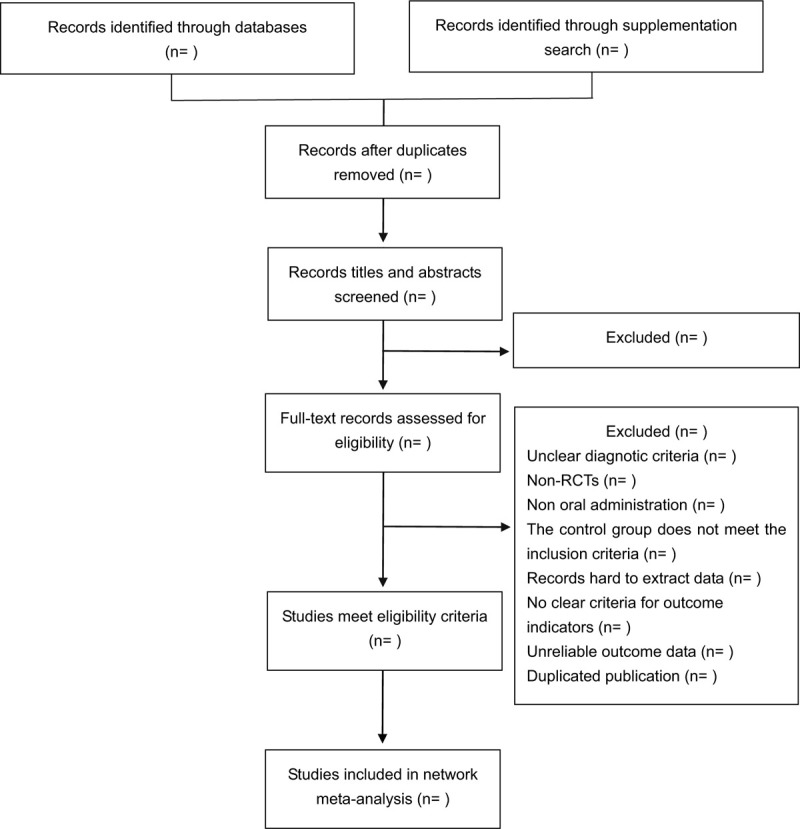
Flow diagram of the study selection.

We will import all retrieved studies into EndNote (version X9, Thomson, Philadephia, PA/USA) and then remove any duplicates. Two researchers (YH and RX) will first independently scan the title and abstract then the full articles will be read when the abstracts lack the information. The articles will be screened according to the pre-established inclusion and exclusion criteria. Any disagreement will be resolved through discussion or underwent third-party adjudication. The flow diagram of literature screening is shown in Figure 1.

### 2.6 Data extraction and management

Two researchers (YH and RX) will independently extract the data according to the following scheme, and the inconsistencies will be reviewed and corrected.

#### 2.6.1 Literature Basic Information

Authors, location, and publication year.

#### 2.6.2 Subject characteristics

Number and gender distribution of patients in the treatment group and the control group. IBS severity, IBS subtypes, race, sex, age, and IBS diagnostic criteria.

#### 2.6.3 Intervention

Treatment method, control treatment, duration, and follow-up time at post-treatment.

#### 2.6.4 Assessment of risk of bias

We will extract relevant information according to the bias evaluation table listed in Cochrane

#### 2.6.5 Outcome measures

All data related to outcome indicators will be extracted.

### 2.7 Assessment of risk of bias

The risk of bias for each study included will be assessed independently by 2 researchers (YH and RX) on the basis of the Cochrane risk-of-bias tool.^[26]^ Six items are included and focused: random sequence generation, allocation concealment, blinding (participants, researchers, and assessors of outcomes), incomplete outcome data, selective reporting, and other bias. Any disagreement will be resolved by consensus or underwent third-party adjudication.

### 2.8 Data analysis

The conventional pair-wise meta-analysis of the direct comparison will be performed using Stata software Stata 14.0 (Stata Corporation, College Station, USA). Data were pooled using a random-effects model because the random effects model will give a more conservative estimate of the precision of the effects in IBS treatment. For dichotomous data, we will use relative risk (RR) with 95% confidence intervals (CI) as the effect measure, and the effect measure of the continuous outcome will be expressed as standardized mean differences (SMD) with 95% CI. Due to the different experimental methods and measuring tools, the existence of heterogeneity between studies is inevitable. Chi-squared test and I^*2*^ tests are determined to evaluate the level of heterogeneity between each paired comparison. The significant degree of heterogeneity will be considered where I^*2*^ is >50% and the Chi-squared test with a *P* < .10.^[27]^ If obvious heterogeneity is found between studies, sensitivity, and subgroup analysis will be applied to examine the source of heterogeneity. If quantitative synthesis is not appropriate, describe the type of summary planned.

Various interventions to treat IBS will be drawn into a network diagram to show direct and indirect comparison. Meanwhile, a funnel plot was drawn for qualitative judgment of publication bias. If the graph was roughly symmetrical, it was considered that there was no publication bias. We will use Stata software (StataV.14.0, StataCorp) to accomplish the network diagram and funnel plot. We will use WinBUGS, version 1.4.3 (Cambridge, United Kingdom) with a Bayesian hierarchical random-effects model to conduct the NMA. To check the consistency of the NMA, the node-split method will be used to determine the location of the inconsistency between direct and indirect treatment effects.^[28]^ The *P* > .05 is considered to be no statistically significant between direct and indirect comparisons, which means the consistency model will be used for NMA analysis; otherwise, the inconsistency model will be conducted.^[28]^ Factors that lead to inconsistency will be discussed later. Finally, we will use the ranking probabilities plots to compare the effects of various interventions.

### 2.9 GRADE quality assessment

We will use the GRADEprofiler version 3.6.1 (GRADE Working Group) to assess the quality of evidence, the confidence in each network will be divided into 4 levels including high, medium, low, and extremely low.^[29]^

## 3 Discussion

Since the importance of intestinal microbes has been widely discussed, more and more attention has been paid to the therapeutic strategies targeting intestinal flora. These 5 therapies (Probiotics, prebiotics, antibiotics, CHM, and FMT) have the commonality of producing therapeutic effects by affecting patients’ intestinal flora, and have been widely used in actual clinical and proved effective. Currently, there has been a study systematically evaluate the effectiveness and safety of probiotics, prebiotics, and antibiotics in the treatment of IBS. However, the content is not comprehensive enough to include fecal bacteria transplantation, a promising new therapy and neither does CHM. Although the mechanism of CHM in IBS has not been determined, it has been widely confirmed that CHM can improve IBS symptoms by regulating the intestinal flora of patients.^[30,31]^ At present, there is a lack of NMA to indirectly compare these 5 treatments. The existing systematic reviews or meta-analysis rarely discuss CHM together with other micro-ecological adjustment drugs, which is obviously 1-sided. Based on this, this study indirectly compared the efficacy and safety of 5 different treatment methods mentioned above for IBS with the method of NMA and selected the optimal scheme. This result can provide clinicians with an evidence-based medical decision-making basis.

## Author contributions

**Data selection, extraction, and bias analysis:** Ying He, Rui Xu, Wang Wei, Xiaoyu Hu. ”

**Database retrieval:** Ying He, Jie Zhang.

**Investigation:** Ying He, Jie Zhang.

**Methodology:** Ying He, Rui Xu, Wei Wang.

**Software:** Jie Zhang.

**Supervision:** Xiaoyu Hu.

**Writing – original draft:** Ying He.

**Writing – review & editing:** Ying He, Xiaoyu Hu.
